# MD-ALL: an Integrative Platform for Molecular Diagnosis of B-cell Acute Lymphoblastic Leukemia

**DOI:** 10.21203/rs.3.rs-2798895/v1

**Published:** 2023-04-14

**Authors:** Zhaohui Gu, Zunsong Hu, Zhilian Jia, Jiangyue Liu, Allen Mao, Helen Han

**Affiliations:** City of Hope; City of Hope; City of Hope; City of Hope; City of Hope; City of Hope

## Abstract

B-cell acute lymphoblastic leukemia (B-ALL) consists of dozens of subtypes defined by distinct gene expression profiles (GEPs) and various genetic lesions. With the application of transcriptome sequencing (RNA-seq), multiple novel subtypes have been identified, which lead to an advanced B-ALL classification and risk-stratification system. However, the complexity of analyzing RNA-seq data for B-ALL classification hinders the implementation of the new B-ALL taxonomy. Here, we introduce MD-ALL (Molecular Diagnosis of ALL), a user-friendly platform featuring sensitive and accurate B-ALL classification based on GEPs and sentinel genetic alterations.

In this study, we systematically analyzed 2,955 B-ALL RNA-seq samples and generated a reference dataset representing all the reported B-ALL subtypes. Using multiple machine learning algorithms, we identified the feature genes and then established highly accurate models for B-ALL classification using either bulk or single-cell RNA-seq data. Importantly, this platform integrates the key genetic lesions, including sequence mutations, large-scale copy number variations, and gene rearrangements, to perform comprehensive and definitive B-ALL classification. Through validation in a hold-out cohort of 974 samples, our models demonstrated superior performance for B-ALL classification compared with alternative tools.

In summary, MD-ALL is a user-friendly B-ALL classification platform designed to enable integrative, accurate, and comprehensive B-ALL subtype classification.

## Introduction

B-cell acute lymphoblastic leukemia (B-ALL) is the most common pediatric cancer and remains a leading cause of childhood cancer death^1^. As a highly heterogeneous disease, B-ALL consists of dozens of subtypes with distinct gene expression profiles (GEPs) and constellations of genetic alterations^2^. With the application of transcriptome sequencing (RNA-seq), multiple novel B-ALL subtypes have been identified harboring recurrent genetic lesions and distinct GEPs^3–5^. The current WHO Classification (5th edition) of Hematolymphoid Tumors (WHO-HAEM5)^6^, along with the International Consensus Classification of Myeloid Neoplasms and Acute Leukemia (ICC)^7^, recognize a total of 11 and 26 molecular subtypes of B-ALL, respectively. While the treatment of B-ALL is moving toward the precision medicine era, it is critical to apply these granular subtypes in clinics to optimize the tailored therapies^8, 9^. Currently, clinical diagnosis and classification of B-ALL is still dominated by a combination of multiple assays such as flow cytometry, fluorescence *in situ* hybridization (FISH), cytogenetic karyotyping, and panel-based sequencing assays^10, 11^. The data generation and analysis using these platforms are time-consuming, expensive, and error prone. More importantly, they are inadequate to identify the subtypes defined by cryptic genetic lesions (e.g., *DUX4*^*3*^ and *MEF2D*^*5*^ rearrangements) or the ones primarily defined by GEPs (e.g., Ph-like^12^, ETV6::RUNX1-like^13^, and PAX5-altered^14^).

With rapid progress in understanding the genetic diversity of B-ALL in recent years, updating clinical test assays accordingly has become a challenging task. Alternatively, applying RNA-seq for clinical diagnosis of B-ALL subtypes has been investigated by multiple institutions and led to encouraging outcomes^15, 16^. With its easy-to-follow protocol and multiple layers of information, RNA-seq is poised to revolutionize the classification of B-ALL in both research and clinical settings. However, bioinformatics analysis of RNA-seq data to extract both the sentinel genetic lesions and the GEP signatures for classification is still highly challenging. Although a few bioinformatics tools have been developed for this purpose^17–19^, they solely rely on GEP for B-ALL subtyping. In this study, we present MD-ALL (Molecular Diagnosis of Acute Lymphoblastic Leukemia), a user-friendly bioinformatics platform that integrates genetic and transcriptomic features from RNA-seq to provide integrative, accurate, and comprehensive B-ALL subtype classification.

## Materials And Methods

### RNA-seq datasets

To establish the training and validation cohorts, we collected raw RNA-seq datasets of 3,005 non-duplicate (according to sample ID) B-ALL samples from multiple published studies^2, 5, 14, 20–27^. Additionally, we inferred the genetic relationship of the enrolled samples using the KING toolkit^28^ based on the genotype of variants called from RNA-seq. We identified twenty pairs of samples as potential duplicates or related, and then removed the ones with relatively lower sequencing coverage. From the remaining 2,985 samples, we further excluded samples with low coding region coverage (< 15% at 30-fold) or low B-cell ratio (< 30%; estimated by the CIBERSORTx^29^; see Methods below). Eventually, 2,955 B-ALL samples with high quality RNA-seq data were kept as the primary dataset for this study (**Supplementary Table 1**).

### RNA-seq data analysis

The raw RNA-seq data were analyzed using a uniform analysis pipeline described in our previous work^14, 21^. In brief, the sequencing reads were aligned to human genome reference GRCh38 using the STAR package (v2.7.6a)^30^. Gene annotation downloaded from the Ensembl database (v102; see URLs) was used for STAR mapping and the following read count evaluation. Then the Picard (v2.26.11; see URLs) was used to mark duplicates and generate the final bam files.

#### Gene expression level evaluation.

Read count per gene was calculated by HTSeq^31^ and FeatureCount^32^, the two most popular tools for this purpose. Then gene expression level was normalized by the variance stabilizing transformation (VST) algorithm in the DESeq2 package^33^. With the VST gene expression data, R packages Rtsne and umap were used to map the samples to 2-dimential t-Distributed Stochastic Neighbor Embedding (tSNE) and Uniform Manifold Approximation and Projection (UMAP) plots using the top variable genes (based on median absolute deviation). The ComBat function in the sva R package^34^ was used to correct the batch effects introduced by different library preparation kits and sequencing lengths (**Supplementary Fig. 1**).

#### Digital deconvolution of bulk GEP data.

To establish a GEP reference for annotating the primary blood cell types, we reanalyzed public single-cell RNA-seq (scRNA-seq) data of 166K cells obtained from eight healthy individuals used in the 1-Million Immune Cells Project (see URLs). Through stringent quality control, we established a GEP reference composed of over 10K cells representing 20 distinct cell types. To distinguish detailed differentiation stages of B cells, the annotation includes common lymphoid progenitors (CLP), pro-B1 (early pro-B), pro-B2 (late pro-B), pre-B1 (large pre-B), pre-B2 (small pre-B), immature B, mature B, and plasma cells. With the single-cell GEP reference, we used the CIBERSORTx^29^ to digitally deconvolute the bulk GEPs of B-ALL samples and delineate the composition of different cell types. The collective amount of B-lineage cells (pro-B1 to mature B) deconvoluted from the bulk samples were used to estimate leukemic cell ratios.

#### Mutation detection from RNA-seq.

The short sequence mutation including single nucleotide variants (SNVs) and insertions/deletions (Indels) were called from RNA-seq by following the best practice workflow from the GATK forum (see URLs) as we reported before^14, 21^. In brief, the bam files were processed by the SplitNCigarReads module of GATK (v4.2.2) to Splits reads that contain Ns in their cigar string. MuTect2 and HaplotypeCaller modules were used to call SNVs and Indels afterwards. The variants reported in the dbSNP (v152) and gnomAD (v3.1) databases as common single nucleotide polymorphisms (SNP; population minor allele frequency ≥ 1%) were removed. Then the remaining mutations were annotated to gene regions by VEP^35^ (v103). For B-ALL subtyping, the analysis was focused on a few signature mutations such as *PAX5* P80R and other *PAX5* mutations, *IKZF1* N159Y, and *ZEB2* H1038R. To further assist B-ALL subtyping, other signature mutations in gene *FLT3*, *IL7R*, *JAK1*, *JAK2*, *JAK3*, *KRAS*, *NRAS*, *PTPN11*, *NF1*, *IKZF3*, and *TP53* recorded in the COSMIC somatic mutation database (see URLs) were also reported.

#### Fusion calling from RNA-seq.

CICERO^36^ (v0.3.0p2) and FusionCatcher^37^ (v1.33) were used as they can sensitively identify gene rearrangements involving highly repetitive regions such as the immunoglobulin heavy chain (*IGH*) locus. Since CICERO analysis may take a long time if the input bam files contain too many reads, we capped the bam files to 50 million reads for CICERO fusion calling. Normally, CICERO and FusionCatcher report dozens or even hundreds of fusions, but most of them are false positive. Therefore, we manually curated all the reported fusions to identify the reliable ones. Due to the complexity of *DUX4* rearrangements, a few of them were rescued through manual inspection of aligned reads in the IGV browser^38^.

#### Copy number variation (CNV) and iAMP21 calling from RNA-seq.

With read counts and SNVs called from RNA-seq, the RNAseqCNV package^39^ was used to detect chromosomal level CNVs. The gender information of the samples was also inferred by RNAseqCNV. Besides standard CNV analysis, RNAseqCNV also provides visualization results that can be used to identify intrachromosomal amplification of chromosome 21 (iAMP21) genetic lesions.

#### GEP-guided detection of genetic lesions.

We detected and validated genetic lesions by using the expression level of specific genes or the overall GEPs. First, we compiled a list of candidate mutations and gene rearrangements that are signatures of different B-ALL subtypes. Then, we identified the genetic lesions that are consistent with the GEP features. For example, *CRLF2* rearrangements are associated with *CRLF2* overexpression, while *DUX4* rearrangements are expected in DUX4 subtype defined by GEP. Similarly, GEP-defined PAX5 P80R subtype indicates both *PAX5* P80R mutations and secondary *PAX5* alterations.

### Ancestry inference from RNA-seq

The ancestral background of enrolled samples was estimated using the iAdmix package^40^, with the genotype of SNPs from the 1000 Genomes Project populations, which include European, African, Native American, East Asian, and South Asian, used as the reference^41^. The genetic ancestral compositions of the test samples were quantified and then used to determine each ethnic group as described in previous reports^42^.

### Construct the GEP reference of B-ALL subtypes

Through integrative analysis of driver genetic lesions and GEPs, the enrolled 2,955 B-ALL samples were classified into 26 molecular subtypes, with 19 having distinct GEP features (S**upplementary Table 1**). To construct a GEP reference for B-ALL classification, we performed iterative sample selection using the PhenoGraph clustering^43^ and k-nearest neighbor (KNN) analysis of two-dimensional UMAP to identify the samples with stable and correct GEP clusters. In addition, the major subtypes with highly distinct GEPs, such as ETV6::RUNX1, KMT2A, DUX4, TCF3::PBX1, and MEF2D, were further trimmed to keep the sample size of training vs. test cohort as around 2:1.

### GEP feature gene selection

Since the GEP reference cohort is not evenly distributed across different B-ALL subtypes, generic feature selection algorithms may favor the features of the major subtypes. To overcome this, cohorts with same sample size per subtype were generated by subsampling major subtypes and artificially constructing additional samples for minor ones using the SMOTE algorithm^44^. Eight different samples sizes (n = 10, 25, 50, 75, 100, 150, 200, and 250) per subtype were used to evaluate whether the feature genes can be stably identified. Then Boruta, a random-forest-based feature selection algorithm^45^, was used to identify the genes confirmed as contributing features for distinguishing different subtypes. Furthermore, to accommodate both mRNA and total RNA-seq libraries, only the protein-coding genes were considered for feature selection.

### GEP-based B-ALL classification model

Using the feature genes and reference cohort described above, two GEP-based B-ALL prediction models were constructed: 1. support vector machine (SVM) classification. Among multiple machine learning algorithms, we observed that SVM performed the best. The reference samples from the 19 distinct subtypes were analyzed by SVM to train a prediction model using different numbers of feature genes (ranging from 100 to 1,058 genes in 11 rounds, with 100 as the interval). 2. PhenoGraph clustering^43^. PhenoGraph is a clustering algorithm originally developed to identify and partition cells into subpopulations using high-dimensional single-cell mass cytometry data. Here it was applied to cluster the test samples with the reference cohort using different numbers of feature genes as described above for B-ALL classification. Since SVM and PhenoGraph models do not provide confidence score for classification, MD-ALL applies the 11 rounds of prediction using different numbers of genes to quantify the prediction reliability. A subtype is reported if the confidence score is above 0.5.

### Integration of genetic lesions and GEP features

GEP-based subtype prediction and key genetic lesions identified from RNA-seq were integrated to assist definitive classification of B-ALL subtypes. For example, if the genetic lesions and GEP predictions point to the same subtypes, a highly reliable classification will be achieved. However, if GEP-based subtyping gives ambiguous prediction score or it is not consistent with the driver genetic lesions, a knowledge-based decision-making is needed. For example, samples with both *BCR::ABL1* fusion and hyperdiploid karyotype should be classified as Ph (BCR::ABL1) subtype, regardless of the GEP prediction. A detailed description of integrating GEP-based prediction and sentinel genetic lesions for B-ALL classification is summarized in Table 1.

### scRNA-seq analysis and B-ALL classification

scRNA-seq reads were analyzed by the Cell Ranger (v6.0.1) pipeline using the human reference genome GRCh38. Genes expressed in at least 5 cells were retained, as were cells with a minimum of 200 expressed genes and less than 10% mitochondrial reads. Cells with gene counts exceeding the median plus 3 median absolute deviation of gene number were considered outliers and removed. Doublet cells identified by the DoubletFinder^46^ R package were also excluded. The Seurat^47^ (v4.0.5) was used for gene expression normalization and variable gene selection. With the GEP reference of blood cell types and B-ALL subtypes described above, the SingleR package^48^ was used to annotate the cell type and B-ALL subtype for each cell.

## Results

### Characteristics of the RNA-seq cohort

**Figure 1:**
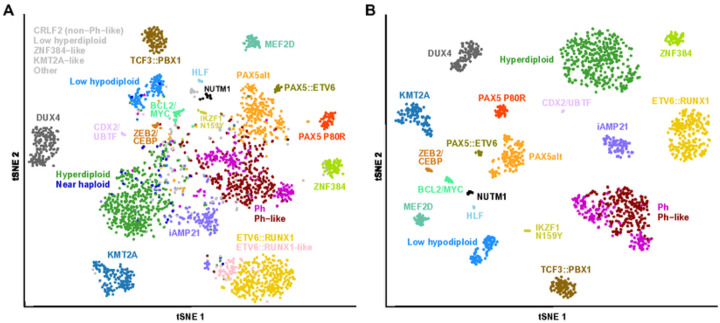
Gene expression profiles (GEPs) of B-ALL subtypes. The tSNE plots display the GEP distribution using 1,058 signature coding genes identified from referenceB-ALL subtypes (see Methods). GEPs are derived from bulk RNA-seq data, with each dot representing an individual sample. A perplexity parameter of 10 was used in tSNE analysis to better visualize the minor subtypes. B-ALL subtypes are color-coded and annotated, while less recognized ones such as CRLF2 (non-Ph-like), Low hyperdiploid, ZNF384-like, KMT2A-like, and unclassified are shown in grey. A. tSNE plot of 2,955 B-ALL samples, which represents the total cohort of this study. B. tSNE plot of reference samples (n=1,821) from 19 B-ALL subtypes with distinct GEPs. For GEP-based classification, Ph and Ph-like are combined one Ph/Ph-like group.

In total, 2,955 B-ALL samples with high-quality RNA-seq data were included in this study (**Supplementary Table 1**). This cohort comprises 67.8% pediatric and 28.4% adult cases from different racial/ethnic backgrounds, with a relative higher proportion of male patients (56.1%) (**Supplementary Fig. 2**). Through manual curation of the genetic lesions, 3,304 gene rearrangements, 2,979 sequence mutations, and 95 *FLT3* internal tandem duplications (ITDs) were identified (**Supplementary Tables 2–4**). Subsequently, sentinel gene fusions and mutations were used to facilitate B-ALL classification (**Supplementary Tables 5 and 6**). Through integration of genetic lesions and GEP-based predictions, the cohort was classified into 26 molecular subtypes (Fig. 1A). In summary, this well-curated large cohort encompasses all the reported B-ALL subtypes across different age groups, genders, and racial/ethnical backgrounds, making it an excellent resource for constructing and evaluating B-ALL subtype prediction models, as well as advancing our understanding of the genetic and transcriptomic features of each B-ALL subtype.

### High accuracy of GEP-based B-ALL classification by MD-ALL

To generate the GEP reference for subtype prediction, 1,821 samples confirmed by sentinel genetic lesions and stable GEP clusters were selected as the training cohort, representing the 19 B-ALL subtypes with distinct GEPs (Fig. 1B). Using this GEP reference cohort, 1,058 feature genes were consistently confirmed by the Boruta algorithm in eight SMOTE-resampled cohorts (**Supplementary Table 7**). Each feature gene was assigned an importance score by Boruta, which was used to rank their significance for distinguishing different subtypes. Based on the reference cohort and selected feature genes, MD-ALL employs SVM and PhenoGraph algorithms to predict the subtypes of the test samples. Considering that the user-provided test RNA-seq data may use different library preparation strategies and the sample size may not be suffi cient for reliable batch effect correction, our prediction models were evaluated using the test samples’ GEP data without batch effect correction as well.

**Figure 2:**
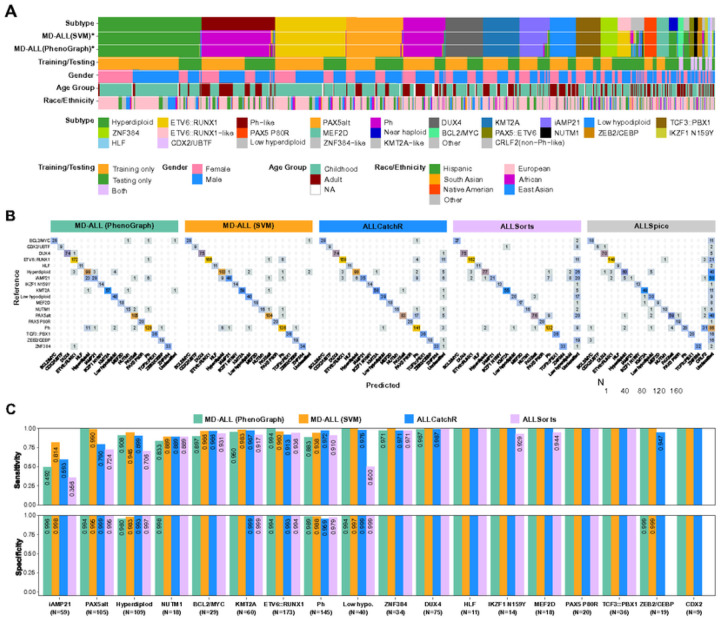
High accuracy of B-ALL subtyping with MD-ALL. A. A heatmap showing the study cohort (n=2,955) highlights B-ALL subtypes and metadata. Each column represents a sample. Two GEP-based subtype prediction models, SVM and PhenoGraph, were established within MD-ALL. *Phenocopy subtypes are identified by their similar GEPs to their corresponding canonical subtypes and are thus annotated with the same colors. For the training/testing annotation, leave-one-out validation was used to evaluate the prediction for minor subtypes, which made samples in these subtypes as both training and testing data. Gender information was inferred using the RNAseqCNV, while race/ethnicity information was determined by the iAdmix package (see Methods). B. A confusion matrix compares subtype predictions made by MD-ALL and alternative tools. The ground-truth subtypes of the 974-sample test cohort are displayed on the left side of each matrix, while prediction results from different models are shown at the bottom. The phenocopy subtypes and their corresponding canonical subtypes are merged for evaluation. MD-ALL, comprising SVM and PhenoGraph models, is compared with ALLCatchR, ALLSorts, and ALLSpice, with ALLSpice showing the largest number of unclassified samples. C. Sensitivity and specificity of GEP-based B-ALL classification. The same test cohort (n=974) described above was used to evaluate all different models. The ZEB2/CEBP and CDX2 (CDX2/UBTF) subtypes are not available in the ALLSorts model. Detailed sensitivity and specificity values are labeled for conditions where they are not 100%. The evaluated sample sizes per subtype are annotated in parentheses.

For the training cohort, 100% accuracy was achieved by both SVM and PhenoGraph algorithms as expected (Fig. 2A). For the test cohort, subtypes with non-distinct GEPs, such as Near haploid, and less recognized subtypes, such as Low hyperdiploid and CRLF2(non-Ph-like), as well as unclassified cases were excluded. To evaluate the performance across different tools, phenocopy subtypes, including Ph-like, ETV6::RUNX1-like, KMT2A-like, and ZNF384-like, were merged with their canonical counterparts to accommodate the different strategies used by different tools for identifying them. Moreover, PAX5alt and Ph-like subtypes are primarily defined by GEP, but their GEP features are less distinct compared with others. To avoid potential bias of evaluating different tools for these two subtypes, only the PAX5alt and Ph-like cases carrying sentinel genetic lesions (i.e., *PAX5* mutation, fusion, or intragenic amplification in PAX5alt, and rearrangements involving kinase activating genes in Ph-like; see Table 1) were kept in the test cohort.

Although this study enrolled a large number of samples, seven minor subtypes have fewer than 30 qualified samples, which include BCL2/MYC (n = 29), PAX5::ETV6 (n = 23), ZEB2/CEBP (n = 19), NUTM1 (n = 18), IKZF1 N159Y (n = 14), HLF (n = 11), and CDX2/UBTF (n = 9). Following the training vs. testing sample size ratio of 2:1 set for the major subtypes, fewer than 10 samples would be left for testing. Therefore, a leave-one-out validation was used to evaluate the prediction models for these minor subtypes, eventually resulting in a test cohort of 974 samples (**Supplementary Table 8**).

Through GEP-based prediction, SVM and PhenoGraph effectively classified 971 and 972 samples into distinct subtypes, respectively, with high overall accuracy achieved in both models (SVM: 96.1%, n = 936; PhenoGraph: 92.7%, n = 903). Despite the high accuracy of both models, SVM surpassed PhenoGraph in discerning multiple subtypes such as iAMP21 and Ph/Ph-like, whereas PhenoGraph demonstrated superior performance over SVM in identifying the ETV6::RUNX1/-like subtype (Fig. 2B&C).

In summary, the GEP-based models in MD-ALL can achieve high classification rate as well as high accuracy for B-ALL classification.

### MD-ALL classification is superior compared with alternative tools

Currently, there are three alternative tools providing the functionality of B-ALL classification, which include ALLSpice^17^, ALLSorts^18^, and ALLCatchR^19^. The subtype prediction by these tools is solely based on GEP; therefore, the comparison with them is restricted to the GEP prediction results of MD-ALL. Additionally, it should be noted that the holdout test cohort of this study partially overlaps with the training cohort of the other tools, since the majority of B-ALL RNA-seq data used in MD-ALL and these alternative tools are from our previous study, which comprises 1,988 B-ALL samples^14^. This overlap may lead to overestimated accuracy for the alternative tools. Additionally, the *PAX5::ETV6* fusion, originally reported as one of the sentinel alterations of PAX5alt subtype^14^, is still considered as PAX5alt by other tools. Therefore, PAX5::ETV6 cases were annotated as PAX5alt when comparing the performance of different models.

In the same test cohort of 974 samples, a higher number of samples remained unclassified by ALLCatchR (n = 36), ALLSorts (n = 142), and ALLSpice (n = 327) when compared to MD-ALL. The overall accuracies were 91.3% (889/974), 81.2% (791/974), and 58.8% (573/974) for each method, respectively, which were significantly lower than those achieved by both models in MD-ALL. When considering only the samples with assigned subtypes, the accuracies of ALLCatchR, ALLSorts, and ALLSpice were 94.8% (889/938), 95.1% (791/832), and 88.6% (573/647), respectively (Fig. 2B). Therefore, the MD-ALL SVM prediction surpassed all other models in terms of classification rate and accuracy. For the MD-ALL PhenoGraph model, when evaluating solely the samples classified by other tools, the accuracies reached 93.7% (879 out of 938 ALLCatchR-classified), 94.8% (789 out of 832 ALLSorts-classified), and 97.1% (628 of 647 ALLSpice-classified), indicating that PhenoGraph is also a highly reliable prediction model for B-ALL subtyping (**Supplementary Table 8**). Among the prediction models, ALLSpice had the lowest number of correctly classified samples (n = 573). Moreover, key B-ALL subtypes, such as Ph-like and ZEB2/CEBP, are not included by ALLSpice, significantly limiting its potential for clinical use. Therefore, ALLSpice will be excluded from further comparisons.

In terms of specificity, MD-ALL (SVM and PhenoGraph), ALLCatchR and ALLSorts demonstrated excellent performance for most subtypes. However, differences were observed in certain subtypes: MD-ALL algorithms outperformed ALLCatchR and ALLSorts in Ph/Ph-like subtype, while ALLCatchR and ALLSorts excelled in Hyperdiploid subtype (Fig. 2C). As for sensitivity, ALLSorts consistently underperformed compared with MD-ALL and ALLCatchR in most subtypes, particularly those with less distinct GEP clusters, such as iAMP21 (35.6%), Low hypodiploid (50.0%), PAX5alt (72.4%), and Hyperdiploid (70.6%). Of note, ALLCatchR performed very well in the test cohort; especially in the Ph/Ph-like group, ALLCatchR surpassed both MD-ALL algorithms in sensitivity (97.2%) at the expense of reduced specificity (95.9%) compared to MD-ALL. As both MD-ALL SVM and ALLCatchR use the SVM algorithm, the high sensitivity levels achieved by these two models are anticipated. However, MD-ALL SVM surpassed ALLCatchR in terms of sensitivity for multiple major subtypes, such as iAMP21 (81.4% vs. 59.3%), PAX5alt (99.0% vs. 79.0%), Hyperdiploid (94.5% vs. 89.9%), ETV6::RUNX1/-like (96.0% vs. 91.3%), ZNF384 (100% vs. 97.1%), and Low hypodiploid (100% vs. 97.5%; Fig. 2C)

In conclusion, when relying exclusively on GEP, MD-ALL demonstrates superior performance over alternative tools in B-ALL classification rate and accuracy, particularly for challenging subtypes such as iAMP21 and PAX5alt.

### Integrative RNA-seq analysis provide reliable and definitive B-ALL classification

Although GEP alone can provide highly accurate B-ALL classifications, sentinel genetic lesions take precedence when GEP results are ambiguous or conflict with the genetic lesions. In this study, 43 near haploid cases were identified based on digital or clinical karyotype. These cases were predicted as hyperdiploid (n = 40) or low hypodiploid (n = 3) by our GEP models. This highlights the importance of integrating GEP predictions with sentinel genetic lesions to accurately determine the subtypes. Moreover, when RNA-seq data is available for evaluating GEP, it would be straightforward to generate genetic lesion information to assist integrative B-ALL classification.

In MD-ALL, users can input raw translocations and sequence mutations for integrative B-ALL classification. Upon reanalysis of 2,955 RNA-seq samples, 96 sentinel gene rearrangements and 587 mutations were identified (**Supplementary Tables 5 and 6**). By integrating GEP and mutation information, MD-ALL calls RNAseqCNV to identify aneuploid subtypes, such as Hyperdiploid, Low hypodiploid, Near haploid, and even iAMP21. Our previous work on RNAseqCNV^39^ demonstrated 100% accuracy in determining aneuploid subtypes, though iAMP21 detection was not mentioned. In this study, we observed high accuracy (35/36) of detecting iAMP21 in B-ALL samples with confirmed iAMP21 status (by SNP array), further broadening the utility of RNA-seq for defining B-ALL subtypes (**Supplementary Fig. 3** and **Supplementary Table 9**).

**Figure 3:**
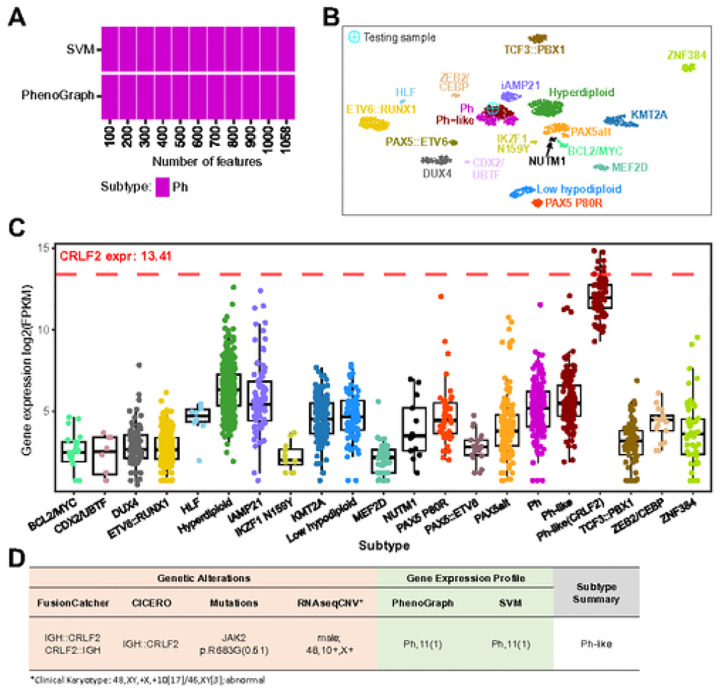
Integrative summary of B-ALL classification by MD-ALL. A. GEP-based subtype prediction by SVM and PhenoGraph models. Different numbers of feature genes are used in the prediction models to evaluate classification robustness. The test sample was consistently predicted as the Ph subtype. B. The test sample is mapped to a predefined UMAP space for visualizing GEP-based classification. The UMAP uses 1,058 features genes. The test sample clusters with the Ph/Ph-like group, which agrees with the SVM and PhenoGraph prediction. C. Expression of a specific gene across different B-ALL subtypes. Ph-like (CRLF2) is shown as a separate group here for confirming *CRLF2* rearrangements. Users can specify a gene to examine its expression for validating genetic lesions (e.g., overexpression of *CRLF2* in *CRLF2* rearranged cases) or potential subtypes. D. Summary of MD-ALL to assist B-ALL classification. The genetic lesions, which include fusions, mutations, large-scale CNVs, will be integrated with GEP-based prediction by PhenoGraph and SVM to assist the classification of the test sample’s B-ALL subtype.

In addition, MD-ALL provides visualization of subtyping results for test sample in SVM and PhenoGraph models using different numbers of genes (Fig. 3A). This visualization aids in assessing the stability of the subtyping results. Furthermore, a UMAP plot of the test sample mapped to the reference cohort using all the feature genes (n = 1,058) offers an insightful overview of the sample’s relationship to the reference (Fig. 3B). As certain gene rearrangements are strongly associated with specific gene expressions, such as *CRLF2* overexpression commonly seen in *CRLF2*-rearranged cases, MD-ALL can display a gene’s expression across all B-ALL subtypes to verify the reliability of specific fusions or subtypes (Fig. 3C). The *JAK2* p.R683G hotspot mutation, known for its high concurrence in *CRLF2*-rearranged cases^49^, further confirms the reliability of the *IGH::CRLF2* fusion. MD-ALL then compiles all input information to assist the final subtype classification. For instance, a sample with an *IGH::CRLF2* fusion and GEP-based Ph/Ph-like prediction, but lacking *BCR::ABL1* fusion, can be definitively classified as Ph-like (Fig. 3D). To facilitate definitive B-ALL classification for all subtypes, MD-ALL incorporates a knowledge-based subtyping guideline that integrates both genetic lesions and GEP features (Table 1).

In summary, MD-ALL offers an integrative RNA-seq analysis solution that evaluates subtyping-relevant information, all derived from the most basic information from RNA-seq data. Combined with specific knowledge in the B-ALL molecular subtyping field, MD-ALL can provide highly reliable and definitive B-ALL classification.

### Distinct B-cell differentiation patterns of B-ALL subtypes

**Figure 4:**
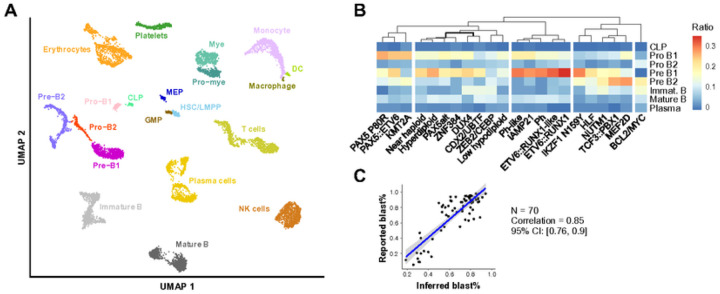
Deconvolution of bulk GEP of B-ALL subtypes A. UMAP of single-cell gene expression reference of the primary blood cell types. Over 10K cells representing 20 primary blood cell types were selected from the 1-Million Immune Cells project (see URLs). B cells are classified into granular differentiation stages, including common lymphoid progenitor (CLP), pro-B1 (early pro-B), pro-B2 (late pro-B), pre-B1 (large pre-B), and pre-B2 (small pre-B). HSC, hematopoietic stem cell; LMPP, lymphoid-primed multipotential progenitor; DC, dendritic cell; Mye, myelocytes; Pro-mye, promyelocytes; GMP, granulocyte-monocyte progenitor; MEP, megakaryocyte-erythrocyte progenitor; NK cell, natural killer cell. B. Heatmap of different B-ALL subtypes and their inferred B-cell differentiation stages. For each subtype, the median value of each B-cell stage is calculated and presented in the heatmap. The Euclidean distance and Ward’s minimum variance clustering method were used to generate the clusters. C. Correlation of digitally inferred and clinically reported blast percentage (blast%). The inferred blast% is estimated by combining B-lineage cells from pro B1 to mature B stages (see Methods). Seventy samples from a cohort provided by the ALLSorts package were used in this analysis.

Using high-quality scRNA-seq data, we compiled a GEP reference consisting of over 10K cells that represent 20 major blood cell types (see Methods; Fig. 4A). Subsequently, we used the single-cell GEP reference to deconvolute the bulk RNA-seq GEP of different B-ALL subtypes (**Supplementary Table 10**). Our analysis revealed that the PAX5 P80R and KMT2A subtypes carry a strong Pro B1 (pre-pro B stage) signature, indicating that they are at the very early stage of B-cell development. By contrast, the BCL2/MYC subtype exhibits a strong enrichment of pre B2 and even immature B cell signatures (Fig. 4B). This suggests that the leukemic B cells are more mature, which is consistent with the observation that *BCL2* and *MYC* rearrangements are more commonly seen in B-cell lymphomas^50^, a malignancy transformed from more mature B lymphocytes. These conclusions agree with clinically reported immunophenotypic features of B-ALL subtypes^23^ as well as other digital deconvolution reports^51^.

To validate the digital deconvolution results, we compared the clinically reported B-cell blast ratio from 70 B-ALL samples and their inferred B-cell ratio by CIBERSORTx, and a high correlation was observed (correlation = 0.85; 95% CI: 0.76–0.9; Fig. 4C and **Supplementary Table 11**). Therefore, digital deconvolution can be used to assess the potential normal cell contamination in bulk samples. In addition, we observed that samples without classified subtypes were enriched with low B-cell ratio (35.9% of 64 samples have < 50% B-cell ratio) compared to those with defined subtypes (3.1% of 2,718 samples have < 50% B-cell ratio). This finding indicates that contamination of normal cells can interfere classification of B-ALL subtypes.

### High sensitivity B-ALL subtyping at a single-cell level

In bulk RNA-seq, it is critical to obtain pure leukemic cells prior to RNA-seq assay to ensure that the GEP represents the disease. However, in clinical settings, patient samples often contain a low proportion of leukemic cells. As a result, B-cell blasts require proper enrichment prior to analysis. Even with B-cell enrichment, samples may still be contaminated by normal B-cell blasts, or contain an inadequate number of enriched cells for bulk RNA-seq.

**Figure 5:**
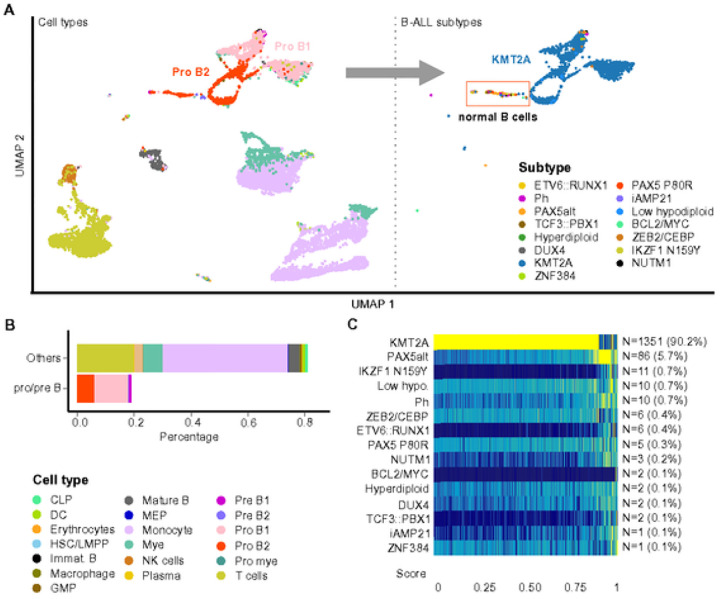
B-ALL subtype classification at a single-cell level A. scRNA-seq of a B-ALL sample at diagnosis shown in a UMAP plot. The abnormally enriched B-cell blasts (pro- to pre-B cells) represent the leukemic cells. With the GEP reference of the B-ALL subtypes, the majority of the B-cell blasts are reliably predicted as KMT2A subtype, which is consistent with the reported subtype. A small cluster (highlighted in a red rectangle) observed with a mixture of different B-ALL subtypes indicates that they are normal B-cell blasts. B. A bar graph shows the distribution of different cell types. Less than 20% of the test sample are B-cell blasts, which could be challenging to be accurately identified as KMT2A subtype based on bulk GEP prediction. C. Heatmap of subtype prediction score shows that over 90% of the B-cell blasts exhibit highly reliable KMT2A GEP signature. Low hypo., Low hypodiploid; CLP, common lymphoid progenitor; HSC, hematopoietic stem cell; LMPP, lymphoid-primed multipotential progenitor; DC, dendritic cell; Mye, myelocytes; Pro-mye, promyelocytes; GMP, granulocyte-monocyte progenitor; MEP, megakaryocyte-erythrocyte progenitor; NK cell, natural killer cell.

To address these challenges, we explored the potential of employing single-cell GEP to identify B-cell blasts (pro- to pre-B cells) using the GEP reference representing major blood cell types (Fig. 4A). After identifying the blast cells, we annotated them to different B-ALL subtypes using the GEP reference compiled from bulk RNA-seq (Fig. 1B). By using public scRNA-seq datasets^52, 53^, we reliably identified multiple B-ALL subtypes, such as KMT2A (Fig. 5), ETV6::RUNX1, Hyperdiploid, and Ph (**Supplementary Fig. 4**), even in samples with blast percentages below 20%. Furthermore, a cluster of B cells was observed with a mixture of different B-ALL subtypes in the KTM2A case, indicating that they are normal B-cell blasts.

In summary, our study highlights the potential of single-cell analysis in the sensitive and accurate detection of leukemic cells and their B-ALL subtypes. With the advent of more cost-effective scRNA-seq platforms and the continual decrease in sequencing costs, single-cell analysis is expected to revolutionize clinical diagnosis of granular disease subtypes.

### MD-ALL: an integrative platform for B-ALL classification

**Figure 6:**
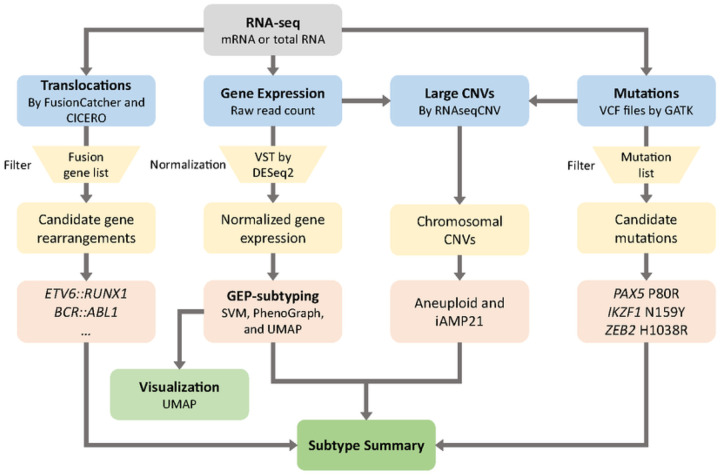
Workflow of integrative B-ALL classification by MD-ALL. MD-ALL accepts three types of standard output from bulk RNA-seq data: translocations (optional; raw output from FusionCatcher and/or CICERO), gene expression (required; read count by HTSeq or FeatureCount), and sequence mutations (optional; VCF files called by GATK). Based on the input data, four aspects of information will be identified: 1) the input translocations are compared with an internal reference to identify signature fusion genes; 2) the gene expression data normalized from raw read count are analyzed by SVM and PhenoGraph to predict the subtype and shown in a UMAP plot; 3) the variants in the provided VCF files are annotated to identify the signature gene mutations; and 4) the gene expression and mutation information are integrated by RNAseqCNV to identify chromosomal CNVs, which will assist the identification of aneuploid and iAMP21 subtypes. Then, a comprehensive subtype summary from the four aspects of information will be integrated to determine the subtypes of the testing samples.

The primary goal of MD-ALL is to provide a user-friendly, one-stop solution for B-ALL classification. To this end, an interactive graphical interface was developed using the R Shiny package, making the tool accessible to users with limited or no computational background. The minimum required input is the raw read count from RNA-seq data. The test sample(s) will be normalized against an internal reference cohort, which consists of 234 samples representing all reported subtypes (**Supplementary Table 12**). This reference cohort was sequenced using various library preparation kits, sequencing lengths, and strandness. Therefore, normalization against this reference helps minimize potential batch effects. The normalized GEPs of test samples are then analyzed by PhenoGraph and SVM models to predict the B-ALL subtypes, as described earlier. Users can also provide raw output of gene rearrangements and mutations to MD-ALL to integrate genetic alterations and GEP information for robust classification (Fig. 6). MD-ALL platform also provides single-cell level B-ALL classification, requiring only the raw read count output from standard scRNA-seq analysis.

Thus, with minimal bioinformatics assistance to generate the raw information of GEP and genetic lesions, users can manage the subsequent analysis using MD-ALL to achieve integrative B-ALL classification and explore gene expression features of different B-ALL subtypes.

## Discussion

In this study, we present the first RNA-seq analysis platform capable of integrating both genetic lesions and GEP features to assist B-ALL classification. Designed with the incorporation of biological knowledge about this highly heterogeneous disease and the informatics characteristics of its various subtypes, the tool aims to offer an intuitive B-ALL classification experience. For most test samples, the integrative analysis will lead to consensus subtypes based on multiple layers of information. Additionally, the platform supplies detailed information for users to review and adjust the results as necessary.

This study is based on one of the largest B-ALL RNA-seq cohorts to establish a GEP reference representing all reported B-ALL subtypes, achieving high accuracy and sensitivity compared with alternative tools. By integrating genetic lesions, which other tools lack, subtypes can be determined more accurately, making this approach more feasible for future translational application in clinical settings.

Using the GEP reference compiled from bulk RNA-seq, we also explored the B-cell differentiation stages of different B-ALL subtypes. Our observations confirmed that certain B-ALL subtypes are blocked at early B-cell progenitor stages, while others progress to more mature stages. Moreover, some subtypes have been observed to have overlapping GEP features, such as iAMP21, PAX5alt, and Ph/Ph-like. Incorporating distinct B-cell differentiation patterns of different subtypes might be beneficial for better separation of these subtypes.

As genomic analysis advances towards single-cell resolution, we have demonstrated the feasibility of using GEP reference derived from bulk RNA-seq for accurate B-ALL classification in multiple subtypes. However, to apply single-cell analysis in clinical settings, all reported B-ALL subtypes still need to be evaluated, possibly with a GEP reference established from scRNA-seq. Currently, generating comparable samples size of single-cell data remains challenging due to technological and cost limitations. Moreover, scRNA-seq is unable to provide as comprehensive transcript abundance as bulk RNA-seq, and different scRNA-seq library preparation kits have been reported with larger batch effects compared with bulk RNA-seq. As a result, bulk RNA-seq remains the optimal platform for generating *bona fide* GEP signatures for each B-ALL subtype.

The classification of B-ALL subtypes using RNA-seq is revolutionizing clinical practice. Moreover, genomic data such as whole-genome sequencing can provide a more comprehensive understanding of genetic alterations, including mutations, CNVs, and structural variations. These results can further confirm the subtypes identified by RNA-seq. Importantly, genetic alterations can further differentiate patients within the same subtypes into more granular prognosis subgroups, making them critical complementary assays for B-ALL classification^54, 55^.

In conclusion, we introduce MD-ALL, a highly sensitive and accurate bioinformatics platform that serves the research and clinical fields for integrative B-ALL classification based on RNA-seq.

## Figures and Tables

**Table 1:** Integrative criteria for B-ALL classification by MD-ALL

Genetic alteration	GEP subtype	GEP feature	Subtype	Note
*BCL2, MYC* or *BCL6* rearrangement	BCL2/MYC	Distinct	BCL2/MYC	The rearrangements can involve genes adjacent to *MYC*
*CDX2* overexpression & *UBTF::ATXN7L3* fusion	CDX2/UBTF	Highly distinct	CDX2/UBTF	*CDX2* overexpression
*CRLF2* rearrangement	Not Ph/Ph-like	Non-distinct	CRLF2(non-Ph-like)	Less recognized subtype
*DUX4* rearrangement	DUX4	Highly distinct	DUX4	*DUX4* gene family overexpression
*ETV6::RUNX1* fusion	ETV6::RUNX1	Highly distinct	ETV6::RUNX1	
No *ETV6::RUNX1* fusion	ETV6::RUNX1	Highly distinct	ETV6::RUNX1-like	Commonly seen with *ETV6* or *IKZF1* rearrangements
*HLF* rearrangement	HLF	Distinct	HLF	*HLF* overexpression
Chromosome number ≥ 51	Hyperdiploid	Distinct	Hyperdiploid	
iAMP21	iAMP21	Less distinct	iAMP21	iAMP21 can be identified by RNAseqCNV
*IKZF1* N159Y mutation	IKZF1 N159Y	Highly distinct	IKZF1 N159Y	
*KMT2A* rearrangement	KMT2A	Distinct	KMT2A	
No *KMT2A* rearrangement	KMT2A	Distinct	KMT2A-like	Minor subtype; Reported with *AFF1* fusion
Chromosome number 47–50	Hyperdiploid	Distinct	Low hyperdiploid	Less recognized subtype
Chromosome number 31–39	Low hypodiploid	Distinct	Low hypodiploid	Commonly seen with *TP53* mutations
*MEF2D* rearrangement	MEF2D	Highly distinct	MEF2D	Commonly seen with chromothripsis around MEF2D
Chromosome number 24–30	Hyperdiploid	Non-distinct	Near haploid	Less frequently with GEP of low hypodiploid
*NUTM1* rearrangement	NUTM1	Less distinct	NUTM1	*NUTM1* overexpression
*PAX5* P80R mutation	PAX5 P80R	Highly distinct	PAX5 P80R	Abnormal *MEGF10* isoform overexpression
*PAX5::ETV6*	PAX5::ETV6	Distinct	PAX5::ETV6	Originally reported as PAX5alt
*PAX5* alteration	PAX5alt	Distinct	PAX5alt¶	Featured with PAX5fusion, mutation, or iAmp, but not deletion
*BCR::ABL 1* fusion	Ph/Ph-like	Distinct	Ph	At least two GEP subclusters observed within Ph group
Non-Ph kinase-activating alteration*	Ph/Ph-like	Distinct	Ph-like	Commonly seen with kinase activating fusions
*TCF3::PBX1* fusion	TCF3::PBX1	Highly distinct	TCF3::PBX1	Rare fusions with *EWSR1* have been reported
*ZNF384* rearrangement	ZNF384	Highly distinct	ZNF384	Also observed in Mixed Phenotype acute leukemia
No *ZNF384* rearrangement	ZNF384	Highly distinct	ZNF384-like	Minor subtype; Reported with *ZNF362* fusion

Note:

If genetic lesions do not agree with GEP-based prediction, genetic lesions determine the primary subtypes, while GEPs guide the decision on the secondary subtypes.

* Gene rearrangements involving *ABL1, ABL2, CSF1R, PDGFRA, PDGFRB, LYN, CRLF2, JAK2, EPOR, TSLP, TYK2, IL2RB, NTRK3, PTK2B, FGFR1, FLT3, DGKH, BLNK*, and *CBL*.

iAmp, intragenic amplification.
